# Comparison of Different Culture Conditions for Mesenchymal Stem Cells from Human Umbilical Cord Wharton’s Jelly for Stem Cell Therapy

**DOI:** 10.4274/tjh.galenos.2019.2019.0439

**Published:** 2020-02-20

**Authors:** Yu Bao, Shumin Huang, Zhengyan Zhao

**Affiliations:** 1 Zhejiang University Faculty of Medicine, Children’s Hospital, Department of Nephrology, Zhejiang, China; 2 Zhejiang University Faculty of Medicine, Children’s Hospital, Clinic of Division of Child Health Care, Zhejiang, China

## To the Editor,

Many recent studies have demonstrated that the umbilical cord is an excellent source of mesenchymal stem cells (MSCs) [1,2,3]. However, in order to use human umbilical cord Wharton’s jelly-derived mesenchymal stem cells (hUC-MSCs) in clinical therapy, a suitable culture procedure for good manufacturing practice-compliant production is mandatory. Nutritional deficiency is the major pathophysiological situation in an ischemic microenvironment in the clinic [4]. Thus, the development of serum-free culture systems is needed [5]. Furthermore, hypoxia is common in vivo in mammals [6]. The average oxygen tension falls to 1% in some cases of pathological ischemia, including fracture hematoma, and in cases of myocardial ischemia [7]. Hence, the investigation of biological characteristics of hUC-MSCs exposed to hypoxic and/or serum-free conditions is of great interest.

In our study, we conducted parallel assays by using four cell groups. For the hypoxic controls, cells from group A (n=10) and group B (n=10) were exposed to 5% CO_2_ and 94% N_2_ in an airtight modular incubator chamber (Billups-Rothenberg Inc., Del Mar, CA, USA). The final oxygen tension was 1%-3% as measured by an oximeter (Oxybaby M+, Witt Technology, Solza, Italy). For the normoxic controls, cells from group C (n=10) and group D (n=10) were placed in an incubator at 37 °C, 5% CO_2_, and 21% O_2_. Cells from group A and group C were expanded in a mixture of Dulbecco’s modified Eagle’s medium and nutrient mixture F-12 (GIBCO, USA) supplemented with 10% fetal bovine serum (GIBCO, USA). Cells from group B and group D were expanded in StemPRO MSC serum-free medium (StemRD, USA). Flow cytometric analysis, differentiation potential, proliferative activities, cell cycle analysis, and apoptosis analysis of these four cell populations were evaluated. We repeated all these experiments 3 times.

**Figure 1:**
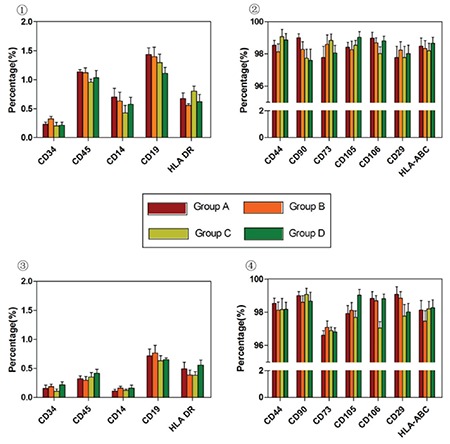
Flow cytometry of hUC-MSC samples [mean percentage ± SD (%)]. 1 and 2: Passage 3; 3 and 4: Passage 6. SD: Standard deviation, hUC-MSC: Human umbilical cord Wharton’s jelly-derived mesenchymal stem cells.

**Figure 2:**
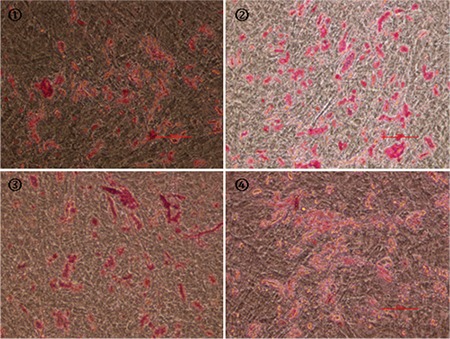
Multilineage differentiation potential of hUC-MSCs. Formation of mineralized matrix was detected by alizarin red S staining. 1: Group A; 2: Group B; 3: Group C; 4: Group D (original magnification: 200x, bar: 50 μm). hUC-MSC: Human umbilical cord Wharton’s jelly-derived mesenchymal stem cells.

**Figure 3:**
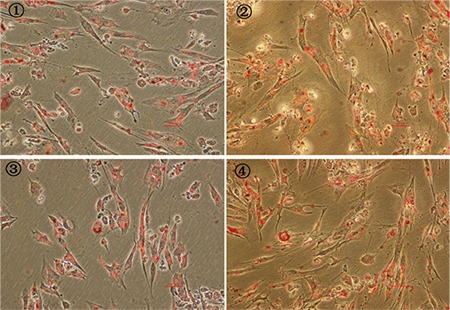
Multilineage differentiation potential of hUC-MSCs. Adipogenesis was confirmed by neutral oil droplet formation stained with oil red O. 1: Group A; 2: Group B; 3: Group C; 4: Group D (original magnification: 100x, bar: 50 μm). hUC-MSC: Human umbilical cord Wharton’s jelly-derived mesenchymal stem cells.

**Figure 4:**
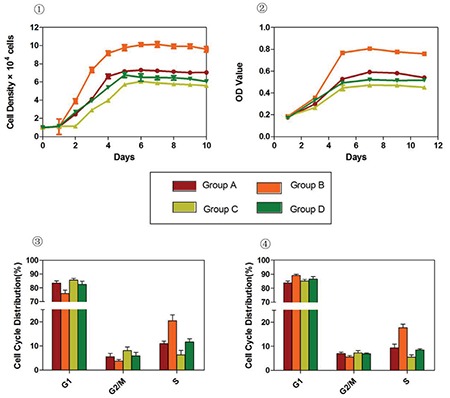
Expansion ability of the four groups. 1: Growth curve at passage 3. 2: MTT assay at passage 3. 3: Cell cycle distribution at passage 3 [mean percentage ± SD (%)]. 4: Cell cycle distribution at passage 6 [mean percentage ± SD (%)]. SD: Standard deviation.

**Figure 5:**
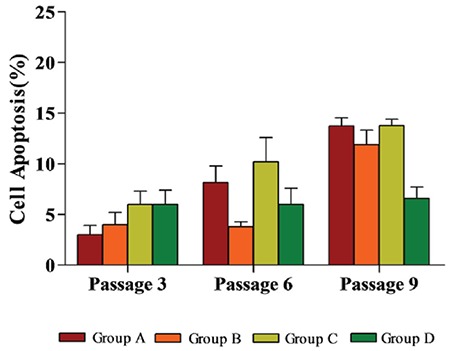
Proportion of apoptotic hUC-MSCs cultured under different conditions (mean ± SD). SD: Standard deviation, hUC-MSC: Human umbilical cord Wharton’s jelly-derived mesenchymal stem cells.

Flow cytometry analysis of MSC-specific surface marker expression showed that hUC-MSCs cultured under four experimental conditions for six passages were positive for CD44, CD73, CD90, CD105, CD29, and HLA-ABC (BD Pharmingen, USA) and negative for CD34, CD45, CD14, and HLA-DR (BD Pharmingen, USA); no significant differences were detected between the four cell populations (Figure 1). This finding indicates that culturing cells under hypoxic and/or serum-free conditions did not induce significant variations in the typical MSC marker expression profile. hUC-MSCs maintained their multilineage differentiation potential in vitro after expansion under various conditions [8]. MSCs from all groups demonstrated a similar osteogenic phenotype, as evidenced by positive staining for alizarin red S (Sigma-Aldrich, USA) and deposits of calcified matrix (Figure 2). In the case of adipogenic differentiation, cells from all groups formed lipid vacuoles detected by oil red O (Sigma-Aldrich, USA). There were no significant quantitative changes among the groups (Figure 3). Thus, the results presented in this report indicate that hypoxic and/or serum-free conditions do not affect the biological characteristics of hUC-MSCs. Under hypoxic and serum-free conditions, hUC-MSCs have higher proliferation according to their growth curves (BD Pharmingen, USA) and MTT assays (BD Pharmingen, USA) than cells grown under normoxic and serum-containing culture conditions, but without more apoptosis (Figures 4 and 5). Taken together, our data indicate that hypoxic and serum-free culture conditions do not influence the major properties of hUC-MSCs. Under hypoxic and serum-free conditions, hUC-MSCs showed higher proliferation, while their apoptosis rate did not increase. This finding is consistent with that of previous reports, which demonstrated enhanced proliferation of bone marrow-derived MSCs (BM-MSCs) under hypoxic or serum-free conditions [9,10]. Therefore, the availability of optimized in vitro conditions, including hypoxia and serum-free media, for hUC-MSC manipulations may have a substantial scientific and clinical impact.
